# Competitive Examinations for Scholarships

**Published:** 1897-05

**Authors:** Richard Grady

**Affiliations:** Baltimore.


AMERICAN JOURNAL

—OF—

DENTAL SCIENCE.

VOL. XXXI. BALTIMORE, MAY, 1897. NO. 1.

ARTICLE I.

### COMPETITIVE EXAMINATIONS FOR SCHOLARSHIPS.

BY RICHARD GRADY, M. D., D. D. S., BALTIMORE.

Read at Tri-Union Meeting, Old Point Comfort, Va., May, 1897.

For years it has been a feature in many (if not all) dental schools to receive beneficiary students, on the payment of half the tuition fees, when they have been recommended by the associations in the several States, or otherwise. Lengthening the time for graduation has increased the expenses incident to three courses of lectures and this, it is said, "works hardships on many young men who are struggling against poverty to obtain an education and are unable to meet the heavy expenses." Time will not be taken to discuss the question of "admitting poor young men to the colleges" or, in another form, "shall poor dental students be encouraged?" nor the statement that "most dental students enter college without proper preparation."

Statistics are not at command, from the 53 institutions teaching dentistry, as to the number of applicants for scholarships; but, applying the information on hand, there are four desiring to attend the dental colleges (as beneficiary students) to one appointed. Some dental schools have furnished, for this paper, the number of students and the number of beneficiaries in 1896-97. If a similar proportion prevailed in the 35 colleges reporting, to the Association of Dental Faculties (varying from 9½ per cent. to 21 per cent. and averaging nearly 14 per cent.) there were from 516 to 1,161 beneficiary students, say more than 750, included in the 5532 matriculates during the year 1895-96. There are aspects of the system which it is impossible to exaggerate. What is the reason for beneficiary students? What is the object?

Previous to the adoption, by the National Association of Dental Examiners and the National Association of Dental Faculties, of the "rule prohibiting colleges from receiving beneficiary students recommended by state boards and associations," the committee on dental education of the Maryland State Dental Association (of which I am chairman) had awarded (early in July, 1896) scholarships in the three dental schools located in Baltimore.

It is the first time that the State Association has insisted upon a standard of education on the part of those entering as beneficiary students. It had been proposed that the examination should be fully equivalent to that for graduation at the Baltimore Polytechnic Institute (similar to the manual training schools of St. Louis, Chicago, Philadelphia) which provides for an adequate preliminary education and skill in the use of instruments; but the committee, anxious to make a beginning, gave an examination so elementary that no exception could be taken to it by anybody with a common school education. No preliminary acquaintance with the theory and practice of dentistry was required, and the examination was confined to three things: (1) Writing a letter; (2) An exercise in dictation; (3) solving four problems in fractions. There were eleven applicants, and three were selected according to their standing under the competitive examination. Respecting the plan, the President of the Maryland State Board of Dental Examiners wrote : "Permit me to add my endorsement, for whatever it may be worth, to the course your committee has taken concerning scholarships to our dental schools. It is certainly a move in the right direction and must be appreciated."

*The examination follows.*

### COMPOSITION.

Write a letter to the committee mentioning

(1) Your name, age, residence.

(2) The dental school in which you desire the scholarship.

(3) What studies you were pursuing when you left school (giving its name and location).

(4) The names and addresses of two persons who would be willing to recommend you.

(5) Why you have decided to study dentistry.

### ARITHMETIC.

(1) If a watch is 18¾ carats fine, how much pure gold is in it ?

(2) Divide 17-18 into two parts, one of which is 2½ times the other.

(3) If I pay 62½cts. a cord for sawing wood 4 feet long into three pieces, how much more should I pay for sawing wood 8 feet long into pieces of the same length ?

(4) A man sold 2-5 of 10-11 of his stock; how many fifths of 10-11 remained ?

### DICTATION.

### THE NEW SCHEDULE.

"The matriculation examination shall be upon the following subjects :

1. *Latin.*—Translation from specified books. Grammatical questions. Easy sentences of English to be translated into Latin.

2. *English.*—Writing from dictation. Questions on English grammar, including parsing, and the analysis of sentences. A short essay to be written on a subject announced at the time of the examination.

3. *Arithmetic.*—As much as is contained in Smith's arithmetic.

4. *Algebra.*—Fundamental principles. Factoring. Fractions. Indices. Surds. Simple equations and quadratic equations with problems involving their use. Arithmetical progression. Geometrical progression.

5. *Geometry.*—First four books of Euclid, with easy exercises.

6. *Physics.*—As much as is contained in Gage's Introduction to Physical Science.

7. One of the following subjects at the option of the candidate :

(a) *Greek.*—Translations from a specified book, with questions in grammar.

(b) *French.*—An examination similar to that in Latin.

(c) *German.*—An examination similar to that in Latin.

(d) *History.*—History of England, or general history as in Swinton's Outlines of the World's History.

(e) *Chemistry.*—As much as is contained in Williams' "Introduction to Chemical Science."

It is recommended that the Board be authorized to secure the consent of the Governor-in-Council to the changes in the curriculum as soon as possible, but that the new schedule shall not go into force until October 1st, 1896.

Resolved, That the matriculation examination for the coming year be in all respects the same as last year."

---

It will be observed that the recommendation of the Association of Dental Examiners adopted by the Association of Dental Faculties prohibits beneficiary students of "state boards and associations" only.

The outlook is that the awarding of scholarships, by the colleges themselves, for exceptional merit, and because of character and promise and not because pecuniary assistance is needed, will take the place of the appointment of (so-called) beneficiary students who have been nominated by personal influence, or on the plea of poverty, or because the candidate is a preacher's son, or becauee the quota from the state in which the applicant resides is full and he is recognized as an actual resident of another state.

Recently the subject has received formal consideration in the Department of Dentistry of the University of Pennsylvania, and examinations for two scholarships will be held next September. I am indebted to Dr. Edward C. Kirk, the Dean of the Dental Faculty, for what follows; and, inasmuch as the subject is of general interest and is treated by an abler hand than mine, what Dr. Kirk has written is quoted at length, in the belief that, when published, it will be suggestive to other dental institutions. He says: "I thought perhaps it might be of interest to you as well as relevant to your subject to send you our circular setting forth the scope of the subjects which form the basis of our competitive examinations for scholarships in dentistry.

"We are constantly applied to by individuals for free scholarships in this department. The basis of the request is usually that the candidate is without means to pay for his tuition. The demands for free scholarships became so numerous and pressing that a committee of the Faculty was appointed to take cognizance of the matter and decide upon some means for meeting the difficulty. After much deliberation it was decided to ask the Board of Trustees to create two University Scholarships in Dentistry to be awarded annually on the results of a competitive examination, the basis of which examination should be the equivalent as that demanded for entrance upon one of the courses in the college department.

"The Board of Trustees have granted the request of the Faculty and the basis of the examination is that set forth in the enclosed circular and is the same as is required for entrance upon either the mechanical, electrical or civil engineering courses of the college department. The competitive examination for these scholarships is open to all applicants without restriction and they will be awarded to the candidates attaining the highest examination results. The Faculty feel that the mere question of inability to pay tuition fees should not be the governing motive in the granting of scholarships but that they should be granted rather upon the basis of merit. Our desire, where we furnish free tuition, is to furnish it only to the very best men who will be a credit to their profession and their alma mater, regardless of the financial question. The only other free scholarships granted in dentistry are those granted under our contract with the Board of Education of this city, numbering two each year which are given to meritorious graduates of our High School. With respect to these men I may say that graduates of the schools named are prepared to pass the matriculate examination of the college department of this University as set forth in the annual catalogue; and where they have attained a certain percentage are admitted upon certificate. No other scholarships are granted in this department. If our annual catalogue is issued before the date of the Union Meeting I shall take the liberty of sending you a copy in order that you may understand more fully the scholarship feature in our school.

Hoping you will have an interesting and successful meeting, I am,

Yours sincerely,

Dr. Richard Grady, Edward C. Kirk.

720 N. Howard Street,

Baltimore.

University of Pennsylvania.—Department of Dentistry.

Subjects which form the basis of the Competitive Examination upon the results of which are awarded the two University Scholarships in Dentistry.

The examinations will be held Tuesday, Sept. 21st, 10 a. m., in College Hall.

### ENGLISH.

No candidate will be accepted in English whose work is notably defective in spelling, punctuation, idiom or division into paragraphs.

A.—(1) Grammar (as in Abbott's *How to Parse*, or Murray's *Advanced Lessons in English Composition, Analysis and Grammar*). (2) Composition.—A short essay, correct in spelling, punctuation, grammar, division by paragraphs, and in expression, written on a subject to be announced at the time of the examination.

B.—Reading. (1) The candidate will be required to present evidence of a *general* knowledge of the subject-matter of the following books set for reading, and to answer simple questions concerning their authors: Shakespeare's *As You Like It*, Defoe's *History of the Plague in London*, Irving's *Tales of a Traveller*, Hawthorne's *Twice Told Tales*, Longfellow's *Evangeline*, George Eliot's *Silas Marner*. (2) A special knowledge of the subject-matter, form and structure of the following works: Shakespeare's *Merchant of Venice*, Burke's *Speech on Conciliation with America*, Scott's *Marmion*, Macaulay's *Life of Samuel Johnson*.

For 1898: A general knowledge of the following works and their authors: Milton's *Paradise Lost*, Books I and II; Pope's *Iliad*, Book XXII; *The Sir Roger de Coverly Papers* in *The Spectator;* Southey's *Vicar of Wakefield;* Coleridge's *Ancient Mariner;* Southey's *Life of Nelson;* Carlyle's *Essay on Burns;* Lowell's *Vision of Sir Launfal.*

A special knowledge of the subject-matter, form and structure of following works: Shakespeare's *Macbeth;* Burke's *Speech on Conciliation with America;* De Quincey's *The Flight of a Tartar Tribe;* Tennyson's *The Princess.*

### HISTORY.

A.—History of the United States (Fiske, Montgomery or Thomas is suggested).

B.—Greek History.—The History of Greece down to the death of Alexander, as contained in Oman's *History of*

*Greece;* with some details concerning the life of the people, as contained either in Oman's *History of Greece*, with Mahaffy's *Old Greek Life*, or in Myer's *History of Greece*.

C.—Roman History.—The history of Rome down to the death of Augustus, as contained in Allen's *History of Rome*, (pages 1-224); with such details concerning the life of the people as are contained in Preston and Dodge's *Private Life of the Romans*.

MATHEMATICS.

B.—Algebra (including factoring, fractions, simple equations and simultaneous equations of the first degree )

C.—Algebra (to the end of quadratic equations, including ratio, proportion, arithmetical and geometrical proportion, arithmetical and geometrical progression, surds and imaginaries, and the binomial theorem for positive entire exponents). (*C does not include B.*)

D.—Plane Geometry (as in the first five books of Chauvenet's or Wentworth's *Geometry*.

E.—Solid Geometry (as contained in Chauvenet's or Wentworth's *Geometry*).

F.—Plane Trigonometry (as in the more elementary portion sof Chaps. I-VI, in Crawley's *Elements of Trigonometry*), and the use of Logarithms.

GERMAN.

A.—Collar-Eysenbach's *German Lessons* (Longer Course), or an equivalent. The candidate's knowledge of grammar will be tested partly by the translation into German of a simple passage of English prose.

B.—At least two hundred (200) pages of modern German prose representing not less than three of the following authors: Benedix, Hauff, Heyse, Riehl and Zschokke.

PHYSICS.

As in Carhart and Chute's, or Gage's, *Elements of Physics*.

